# Cyanotoxins and Cyanobacteria Cell Accumulations in Drinking Water Treatment Plants with a Low Risk of Bloom Formation at the Source

**DOI:** 10.3390/toxins10110430

**Published:** 2018-10-26

**Authors:** Husein Almuhtaram, Yijing Cui, Arash Zamyadi, Ron Hofmann

**Affiliations:** 1 Department of Civil and Mineral Engineering, University of Toronto, 35 St George Street, Toronto, ON M5S 1A4, Canada; maggiecui@napier-reid.com (Y.C.); ron.hofmann@utoronto.ca (R.H.); 2 Civil, Mineral and Mining Engineering Department, Polytechnique Montréal, P.O. Box 6079, Station Centre-ville, Montreal, QC H3C 3A7, Canada; arash.zamyadi@polymtl.ca

**Keywords:** microcystin-LR, anatoxin-a, cyanotoxin, cyanobacteria, harmful algal bloom, accumulation, drinking water treatment

## Abstract

Toxic cyanobacteria have been shown to accumulate in drinking water treatment plants that are susceptible to algal blooms. However, the risk for plants that do not experience algal blooms, but that receive a low influx of cells, is not well known. This study determined the extent of cell accumulation and presence of cyanotoxins across the treatment trains of four plants in the Great Lakes region. Samples were collected for microscopic enumeration and enzyme-linked immunosorbent assay (ELISA) measurements for microcystins, anatoxin-a, saxitoxin, cylindrospermopsin, and β-methylamino-L-alanine (BMAA). Low cell influxes (under 1000 cells/mL) resulted in significant cell accumulations (over 1 × 10^5^ cells/mL) in clarifier sludge and filter backwash samples. Microcystins peaked at 7.2 µg/L in one clarifier sludge sample, exceeding the raw water concentration by a factor of 12. Anatoxin-a was detected in the finished drinking water of one plant at 0.6 µg/L. BMAA may have been detected in three finished water samples, though inconsistencies among the BMAA ELISAs call these results into question. In summary, the results show that plants receiving a low influx of cells can be at risk of toxic cyanobacterial accumulation, and therefore, the absence of a bloom at the source does not indicate the absence of risk.

## 1. Introduction

Harmful algal blooms (HABs) in the Great Lakes region of North America are increasing in frequency and severity and can include toxic cyanobacteria strains [1]. The toxins produced include microcystins, anatoxin-a, saxitoxin, cylindrospermopsin, β-methylamino-L-alanine (BMAA), and aplysiatoxins [2,3,4,5,6]. Since much of the drinking water supply in the Great Lakes region comes from surface water, HABs present an increasing threat to public health.

Previous research has demonstrated that during algal blooms, cyanobacteria can accumulate inside drinking water treatment plants to cause operational problems such as filter clogging, and this may also lead to toxin accumulation to concentrations exceeding those observed in the source water [7,8,9,10,11,12]. There is little information, however, on the potential for such toxic cell accumulation in plants drawing from water bodies that do not experience obvious algal blooms at the water surface. 

The World Health Organization (WHO) provides guidance to drinking water treatment plants to address the risk of HABs [3]. Actions that a plant might take to minimize the threat depend on the magnitude of the bloom at the source, as measured by cyanobacteria cell density (cells/mL), biovolume (mm^3^/L), or chlorophyll-a concentration (µg/L). In general, the absence of evidence of a significant amount of cyanobacteria (e.g., fewer than 2000 cells/mL) indicates that a plant may remain at a basic level of vigilance. When indicators become more pronounced, such as when there are more than 2000 cyanobacteria cells/mL present, the WHO recommends intake optimization to avoid cyanobacteria, increased toxin monitoring, and risk assessments [3]. However, one potential weakness of this approach is that even if cyanobacteria concentrations are at a low level at the intake, any low concentration might result in cyanobacteria cells accumulating inside water treatment plants over time, since treatment plants remove particles from the water and collect them as sludge or inside filter media. If the sludge or filter media is not frequently and effectively removed or backwashed, the concentration of cyanobacteria inside the plant may increase to levels that are significantly greater than in the source water. 

While the increased risk of cyanobacteria and toxin accumulation inside water treatment plants during a bloom event at the source may be intuitively obvious, evidence of whether such accumulation might occur in the absence of a bloom at the source has not, to date, been reported. This is likely due, in part, to the regulatory framework in many jurisdictions, such as in Canada. In Canada, monitoring for cyanobacteria or related toxins in the source water or in treated water is normally triggered by observations of blooms in the source water. In the absence of a bloom, water quality in the plant will typically not be monitored for cyanobacteria or toxins. The assumption that there would not be cyanobacteria or toxins in the plant at problematic levels in the absence of an obvious bloom at the intake has not been tested to date. Given the strong evidence for significant magnification of cyanobacteria concentrations within plants during a bloom event, the same potential for magnification when cyanobacteria are at lower levels at the intake should also be assessed proactively to identify any potential threats to public health. This study, therefore, is the first to monitor systematically four drinking water treatment plants drawing water from the Great Lakes for the potential accumulation of cyanobacteria and their related toxins regardless of the susceptibility of the source water body to HABs. The toxins monitored included microcystins, anatoxin-a, cylindrospermopsin, saxitoxin, and BMAA. 

Cyanobacteria can be quantified using several methods including traditional microscopic enumeration, quantitative polymerase chain reaction (qPCR), and, with more recent technological advances, remote sensing. However, these methods are expensive, time consuming, and require highly trained personnel to manage [13,14]. Furthermore, none can produce results in real time. In contrast, affordable online fluorescence monitoring probes can measure the fluorescence of the photosynthetic pigment phycocyanin, which is representative of the cyanobacteria community, automatically and in real time.

Recently, researchers have explored the use of in situ fluorescence measurements to assess the cyanobacteria risk entering drinking water treatment plants. Fluorescence readings can be correlated with moderate to high linearity to cyanobacteria cell concentration or biovolume [8,15,16,17,18,19,20,21,22], though there are many potential sources of interference [23,24,25,26]. This relationship can be used to calculate a site-specific fluorescence threshold value in raw water to trigger treatment adjustments that improve cyanobacteria removal [8,20]. A statistical interpretation of probe readings may be used to predict the likelihood of exceeding a cyanotoxin threshold [27]. Additionally, fluorescence patterns in settler basins and filters can help to identify the location of cell agglomerations three-dimensionally [11]. This study explored the use of a monitoring probe to produce a fluorescence threshold that corresponds to the WHO framework and further investigated the utility of fluorescence probes when employed across the treatment train, where possible, to provide a qualitative assessment of cell accumulation and breakthrough. 

The precise objectives of this study were, therefore, the following: (1) to determine the extent of cell accumulation in plants despite no obvious algal blooms at the intake; (2) to quantify the concentrations of five cyanotoxins across the treatment train of each plant; (3) to explore the potential impact of a cyanotoxin release due to accumulated cells within the plant; and (4) to assess the value of an in situ fluorescence probe to estimate cyanobacteria biovolume across the treatment train.

## 2. Results and Discussion

### 2.1. Cyanobacteria Accumulation across Treatment Trains

**Figure 1:**
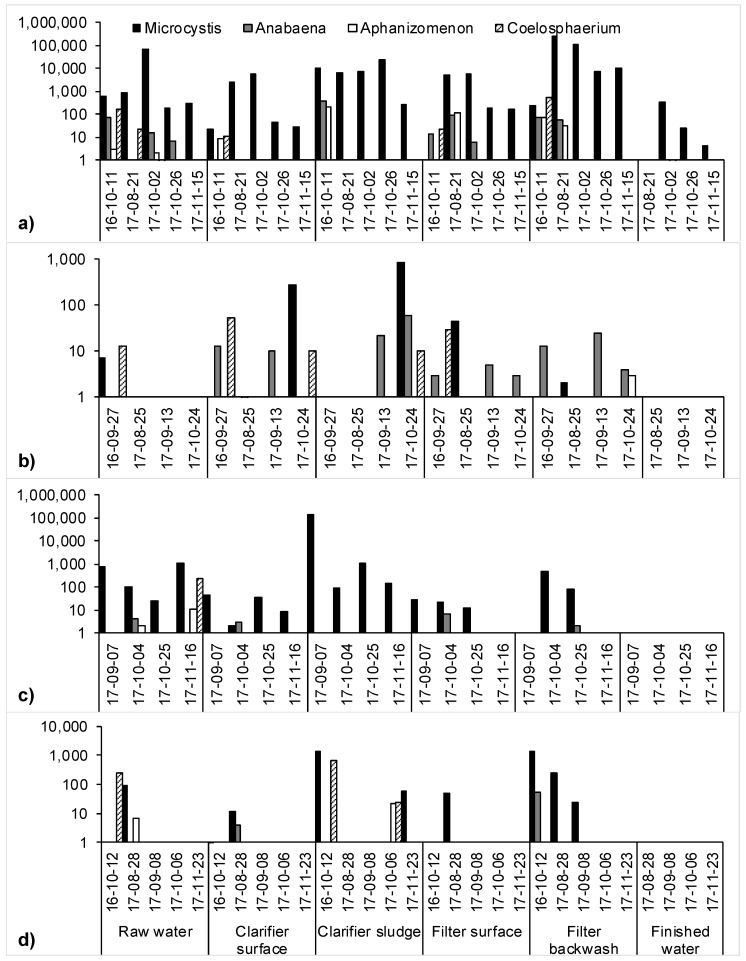
Log cyanobacteria (cells/mL) across (**a**) Plant A, (**b**) Plant B, (**c**) Plant C, and (**d**) Plant D on each of the dates samples were collected (yy-mm-dd).

Cyanobacteria accumulation across the treatment trains was widely observed in this study, demonstrating that monitoring programs considering the raw water alone may be insufficient to capture the complete risk due to cyanobacteria. Over 70% of the filter backwash samples contained a concentration of cells greater than that of the raw water, a proportion greater than any other location measured (Figure 1). Though clarifier sludge samples less frequently contained a concentration of cells greater than the raw water samples, when they did, the magnitude of concentration was greater than that of the backwash samples, on average. Therefore, the filter media after a complete or nearly complete run time (represented by the backwash) and clarifier sludge were identified as the most problematic areas for cell accumulation in this study. A previous study reported the formation of cyanobacteria scums during the algae season on the surface of the clarifiers and filters with a concentration of up to 1.1 × 10^6^ cells/mL [11]. Interestingly, those scums were dominated by Microcystis species as were the majority of samples in this study, so it is unclear why scums formed in those plants but not in the plants in the present study despite comparable cell concentrations in the raw waters (Figure 1). It is possible that local environmental conditions influenced the buoyancy of the Microcystis cells [28,29]. 

Inconsistent results concerning clarification were observed in this study. On 2 October 2017, Plant A experienced nearly 70,000 Microcystis cells/mL in its intake and its upflow clarification process reduced that concentration to approximately 2600 cells/mL. On 21 August 2017, however, the same clarifier at the same plant led to an increase in Microcystis cell concentration from 874 cells/mL in the raw water to approximately 7200 cells/mL in the clarified water (Figure 1a). The clarifier performance was therefore variable in its ability to reduce influent cyanobacteria cells. This suggests a need to optimize clarification for cyanobacteria removal throughout the algae season, such as by regular jar testing.

Accumulation in Plants B and D was generally very low but Microcystis was still the most dominant genus, shown in Figure 1b and Figure 1d, respectively. The sedimentation tank at Plant B undergoes only semi-annual sludge removal, leading to concerns over significant cyanobacteria accumulation. However, three of the four sludge measurements at that plant showed little to no cyanobacteria, while the fourth sampling revealed a concentration of only 900 cells/mL.

Though high concentrations of cells in drinking water treatment plants can cause physical disruptions to the treatment process, an important concern with cyanobacteria is the risk for toxin release. The World Health Organization has established a framework that implies a correlation between toxin risk and the cyanobacteria biovolume or cell counts in the source water [3], and previous studies have noted such correlations [10,30]. In this study, however, there was no observed correlation between toxin concentrations and biovolume nor phycocyanin fluorescence, as shown in Figures S1 and S2 in the Supplementary Information, respectively. 

**Figure 2:**
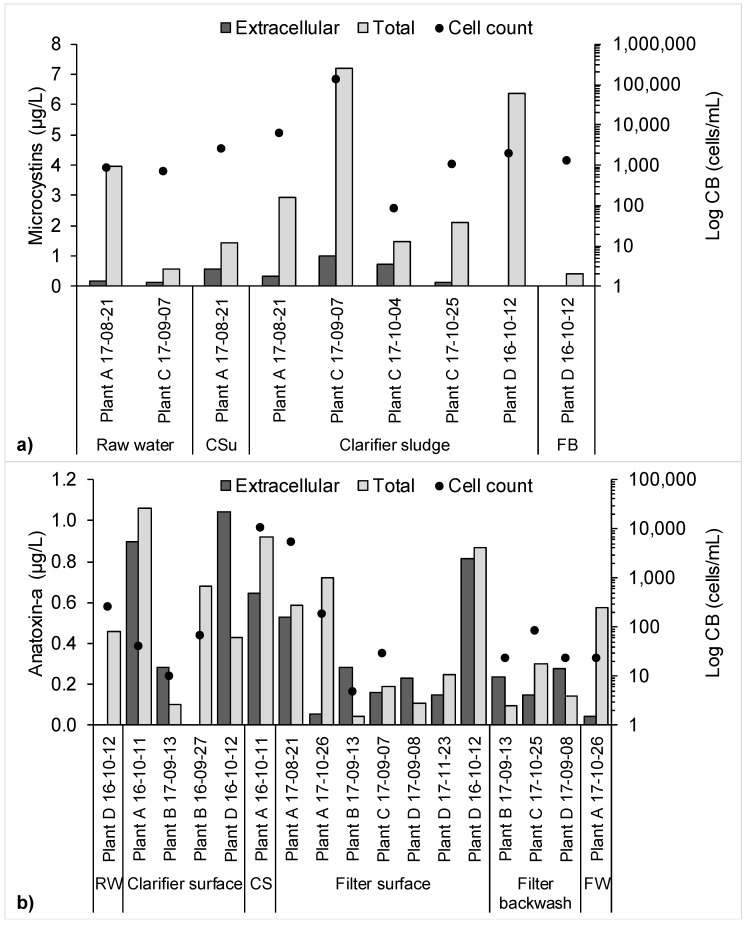
Dissolved and total (**a**) microcystins and (**b**) anatoxin-a detected above the MDL with the corresponding cyanobacteria (CB) cell counts. RW: raw water; CSu: clarifier surface; CS: clarifier sludge; FB: filter backwash; FW: finished water.

Low levels of accumulation (up to 2000 cells/mL) produced intracellular microcystins in the range of 3.8–6.4 µg/L, indicating that these cells had a high cell quota for microcystins (3.3–4.2 pg/cell) (Figure 2a). In contrast, a comparable intracellular microcystins concentration of 6.2 µg/L in a clarifier sludge sample corresponded to a cell concentration of over 100,000 cells/mL, indicating the cell quota in this case was only 0.05 pg/cell. The highest microcystins cell quota calculated was 8.7 pg/cell in a sample containing 87 cells/mL and 0.76 µg/L intracellular microcystins, assuming that the toxins measured were produced solely by the cells enumerated in that sample and evenly among the cells, although in reality other factors such as environmental conditions, bloom growth stage, and interspecific competition affect toxic gene expression [31,32,33]. This demonstrates significant variability in cyanotoxin cell quota [34]. 

**Figure 3:**
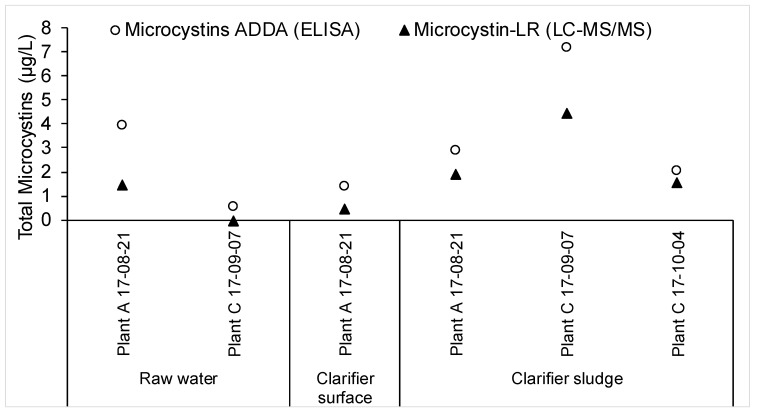
Total microcystins measured by ELISA and total microcystin-LR measured by LC-MS/MS.

The trend of the ELISA results conformed well with that of the LC-MS/MS results for microcystin-LR, though the microcystin-LR concentrations were lower than the microcystins concentrations, suggesting the presence of other microcystin variants in these samples (Figure 3). Microcystins were most frequently found in the clarifier sludge samples across all the sites. The accumulation of microcystins in clarifier sludge might be particularly troublesome for plants that employ upflow clarifiers, such as Plant A. In such a treatment process, there is the additional risk that the sludge blanket could rise over the weir and deliver a concentrated slug of toxins to the filters. Nonetheless, no microcystins were detected in any filter surface, backwash, or finished water sample.

Extracellular anatoxin-a was detected in the absence of cyanobacteria cells, shown in Figure 2b. Anatoxin-a was detected in samples spanning all sites and all stages of the treatment process, and may have been present in one finished drinking water sample at 0.6 µg/L (MDL = 0.24 µg/L) in the intracellular form. The coefficient of variation among the 10 control samples in the ELISA kit wherein anatoxin-a was detected in a finished water sample was 0.11, though that sample failed to produce a response in one of the duplicate measurements. Anatoxin-a is especially challenging to treat for conventional drinking water treatment plants because it is resistant to degradation by chlorine, though granular activated carbon adsorption, employed by Plant C in this study, may be effective [35].

**Figure 4:**
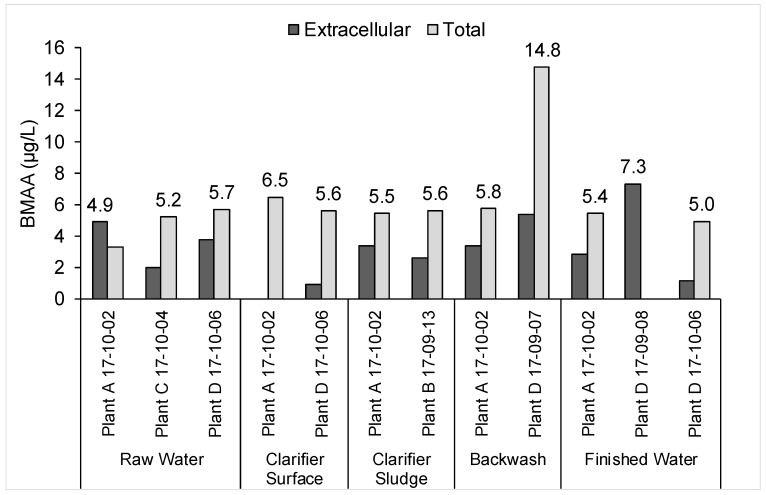
Dissolved and total β-methylamino-L-alanine (BMAA) measurements from two of five ELISA test kits. Data labels are included above the measurements that were above the MDLs.

BMAA was detected above the MDL in 12 samples, as shown in Figure 4. It is worrying that BMAA may have been found in three separate drinking water samples within the range of 5.0–7.3 µg/L because of its potential link to neurodegenerative disorders [36,37]. However, caution should be exercised when interpreting these findings. Two BMAA ELISA kits were used to produce Figure 4, and while recoveries of the calibration standards in the two kits ranged from 62–148%, with an average of 100% recovery, recovery of the control samples ranged from 31–232%, although the concentration of the control samples was 5 µg/L, which is close to the calculated MDLs and would explain the high variability in control recovery. False positives were observed in 5 of the 16 MilliQ blank samples measured among the five kits. Given the mixed results of the BMAA ELISA kit in the literature, it cannot be concluded with certainty that BMAA was present in these samples [38,39,40].

### 2.2. Risk Assessment for the Worst-Case Scenario Toxin Release Event

No exceedances of local drinking water standards were found in this study. There is a theoretical risk, however, that any accumulated cells could lyse and release their toxins, causing a high toxin concentration to break through to the finished water. A simple calculation was therefore performed to estimate the upper-bound risk associated with such a scenario, using the maximum cell concentration of 240,000 cells/mL of Microcystis sp. observed in the filter media of Plant A (Figure 1a). The maximum cell quota for microcystins by *Microcystis* sp. reported by Buratti et al. [34] is 4 pg/cell, however a maximum cell quota of up to 8.7 pg/cell was attributed to cells in this study, see Section 2.1. With 240,000 cells/mL producing microcystins at 8.7 pg/cell all undergoing lysis at once (i.e., the worst-case), this would result in a concentration of 2088 µg/L microcystins entering the chlorine contact chamber. Modelling with CyanoTOX V 2.0 (Hazen-Adams) reveals that a concentration x time (CT) of 128.8 mg·min/L with free chlorine is required to reduce the microcystins to a safer level of 0.3 µg/L. Whereas it is possible that some large-scale municipal drinking water systems can achieve such a high CT value, smaller systems may not, and this would consequently pose a health risk to the consumer. This issue is exacerbated if the cyanotoxin in question is anatoxin-a: the model predicts that the same CT value of 128.8 mg·min/L would result in only 7.4% removal for any concentration of anatoxin-a. 

In reality, however, the toxin concentration coinciding with the sample containing the 240,000 cells/mL in this study was below the detection limit. Toxin release occurs as a result of natural (cell decay) or induced (physical or chemical stress) lysis [41] and is difficult to predict. Additionally, the volume of water surrounding the filter media and carrying the toxins would be significantly diluted in a clearwell. Therefore, it is possible that even in the worst-case scenario for the results of this study, microcystins would be degraded to safe levels under normal operating conditions. 

### 2.3. Use of A Fluorescence Probe for Improved Cyanobacteria Monitoring

Monitoring guidelines of some jurisdictions recommend visual inspection of a source water as a reactive trigger for cyanobacteria prevention strategies. However, it was shown that the presence of an algal bloom does not indicate that a treatment plant is necessarily at risk. Algal blooms were observed on the surface of the source waters of Plant A (Figure 1a) on 21 August and 2 October 2017 and Plant C (Figure 1c) on 7 September and 4 October 2017. In only one of these four instances, however, did the concentration in the raw water intake exceed the WHO Alert Level 1 of 2000 cells/mL, reaching 70,000 cells/mL. Even so, the cell concentration in the clarifier surface, clarifier sludge, and filter surface was reduced to below 8000 cells/mL while the filter backwash contained only 1.6 times the concentration of the raw water, and no cyanotoxins were detected that day. Therefore, the mere presence of a bloom on the water surface does not indicate that cyanobacteria will enter the intake and necessitate preventative actions in a drinking water treatment plant. 

The lack of breakthrough of cyanobacteria into a plant despite a visible bloom on the water surface is presumably due to the depth of the intake, which makes accurate visual monitoring difficult as some cyanobacteria species will form surface scums and not distribute themselves throughout the water column. Conversely, the absence of an algal bloom at the water surface does not indicate the absence of cells at the location of the intake, as previously shown [11]. Therefore, there is a need for a real-time quantification tool to measure cyanobacteria in the actual intake water to drinking water treatment plants. 

**Figure 5:**
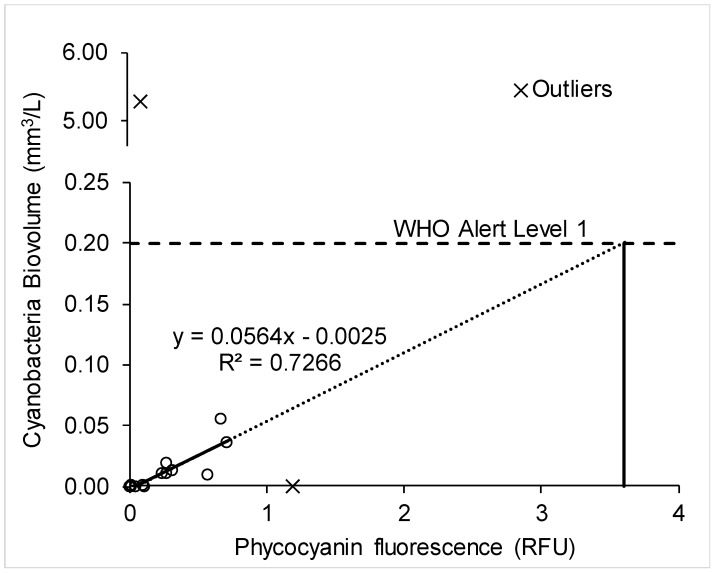
Paired probe data with cyanobacteria biovolume from the raw water samples of all sites.

This study examined the use of a monitoring probe to quantify the biovolume (mm^3^/L) of cyanobacteria entering the treatment plants, as well as to assess its ability to detect cyanobacteria at various locations within the plant. A moderate correlation (*R*^2^ = 0.73, *n* = 13, *p* = 0.0018) was established using paired phycocyanin fluorescence and biovolume data from the raw water samples of all the sites, shown in Figure 5. Previously reported coefficients of determination for the correlation of cyanobacteria biovolume (mm^3^/L) to phycocyanin RFU range from *R*^2^ = 0.41 (*n* = 53) to 0.87 (*n* = 46) [8,12,19,22,24]. Macário et al. [42] found that phycocyanin is a better predictor for biovolume than cell concentration and indeed the correlation with cell concentration in this study was less accurate and not used. 

The moderate correlation between biovolume and fluorescence in the raw water might allow the probe to be used as a surrogate for monitoring biovolume, allowing the World Health Organization framework to be applied in real time. As a theoretical example of how this might work, a threshold phycocyanin fluorescence value of 3.6 RFU was extrapolated to correspond to the WHO Alert Level 1 biovolume of 0.2 mm^3^/L (Figure 5). A probe reading of 3.6 RFU could therefore be used as a trigger level to require toxin analysis across the treatment train, instead of relying on time-consuming biovolume measurements by microscopy. This is a hypothetical example, however, and not truly justified by the data in Figure 5. More data would be required across the biovolume/fluorescence range before such an approach could be used with confidence. 

One further consideration is that the WHO framework does not account for cyanobacteria cell accumulation in drinking water treatment plants. While the 0.2 mm^3^/L Alert Level 1 value is derived from the threshold biovolume that could produce microcystins above the WHO guideline in raw water, a much smaller biovolume can cause significant cell accumulation. For example, a biovolume of only 0.05 mm^3^/L (870 cells/mL) in the raw water sample of Plant A on 21 August 2017 was associated with a biovolume of 18.5 mm^3^/L (240,000 cells/mL) in that day’s backwash sample, as shown in Figure 1a. For a monitoring probe to inform on the breakthrough of cells in a plant, it must be applied across the treatment train. 

**Figure 6:**
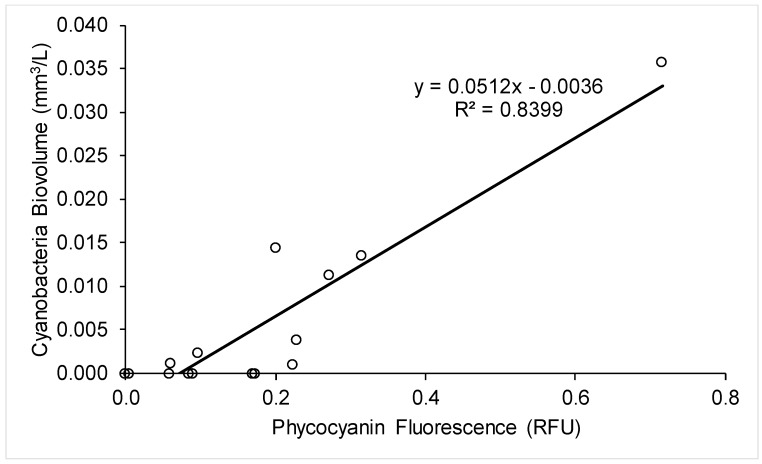
Cyanobacteria biovolume and probe readings from the samples taken across the treatment train of Plant C.

A strong correlation between cyanobacteria biovolume and probe readings was established for samples taken across the treatment train of Plant C (*R*^2^ = 0.84, *n* = 15, *p* = 0.0007), shown in Figure 6. This finding can be used to quantify in real time the breakthrough of cyanobacteria by installing a probe in one or more of the locations in Plant C where this data was collected: the raw water, the clarifier surface, the filter surface, and the finished water. The use of a probe to quantify cyanobacteria at various points along the treatment train could be a powerful tool for operators to optimize treatment reactively where sufficient data can be obtained, but the difficulty of establishing an accurate correlation remains a barrier [20]. 

A similar correlation could not be found for the remaining three sites nor for data pooled from all four sites (*n* = 60, Figure S1, Supplementary Information). The presence of multiple outliers and numerous samples lacking any cyanobacteria but still producing a fluorescence signal are causes for these nonlinearities. Furthermore, by combining paired fluorescence readings to biovolume across the treatment train, an additional source of error may be introduced: specific absorption of phycocyanin varies with phycocyanin concentration and with cell morphology [43]. Phycocyanin concentration in turn varies significantly among species [42], and some species are more readily removed by conventional treatment processes than others, although Microcystis generally dominated the species composition of samples in this study (the species composition in all the samples is shown in Table S1 in the Supplementary Information). 

## 3. Conclusions

This study sought to expand the available information on cell accumulation in drinking water treatment plants with a focus on plants considered to be at little-to-no risk of HABs. Significant cell accumulations of up to 240,000 cells/mL occurred despite low cell concentrations in the raw water (under 1000 cells/mL). Microcystins and anatoxin-a were detected across the treatment trains but did not correlate to cell concentrations. Anatoxin-a was detected in a drinking water sample. This is concerning because if accumulated cells release anatoxin-a, it could have adverse consequences for public health, given that it is resistant to degradation by chlorine. BMAA may have been detected within the plants studied but the results were marred by inconsistencies among the ELISA kits, demonstrating that LC/MS validation is needed to confidently report BMAA concentrations.

These findings illustrate the inadequacy of current monitoring practices that rely on visual monitoring to trigger reactive measures in drinking water treatment plants. The mere presence of a visible bloom on the water surface may not necessarily result in more cyanobacteria at the intake of a treatment plant. Moreover, the low concentration of cells able to cause significant accumulation may not be high enough to impart a change in the appearance of the raw water to the naked eye. As such, measurements from a fluorescence monitoring probe were paired to biovolume to quantify cyanobacteria across the treatment trains. A significant correlation was found using data from the raw water samples of all four sites, but a correlation for samples taken across the treatment trains was found for only one site. The risk for toxic cyanobacterial accumulation may not be identifiable even with a functional online cyanobacteria-monitoring system at the intake as significant accumulation can occur despite a low cell flux. Instead, online monitoring of cyanobacteria across the treatment train can inform utilities of the breakthrough of cyanobacteria at each treatment step, allowing them to make optimizations precisely where they are needed. 

## 4. Materials and Methods

### 4.1. Cell Accumulation Sites

Sampling campaigns were undertaken at four drinking water treatment plants in Ontario, Canada. Samples were collected during the algae season from August–November 2017 as algae are not expected to proliferate until the late summer or early fall. The intakes of the four plants are located in Lake Erie, Lake Ontario, the Bay of Quinte in Lake Ontario, and an inland reservoir, and are labelled Plants A–D, respectively. The two plants drawing water from Lake Ontario and the inland reservoir have a low risk for HABs. The sources of the two plants drawing water from Lake Erie and the bay in Lake Ontario routinely experience blooms, but the depth of their intakes reduces their susceptibility to HAB effects. All four plants employ conventional treatment trains, with minor differences. A summary of the treatment processes of the four plants is presented in Table 1. 

### 4.2. Sampling Procedure

**Figure 7:**
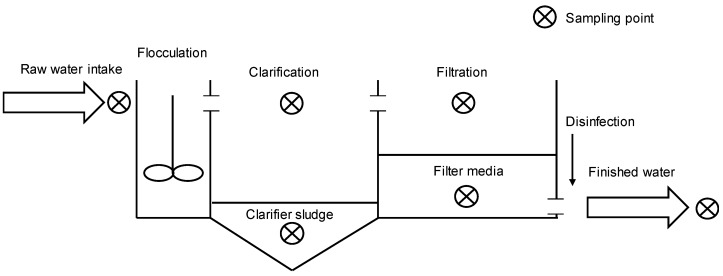
Schematic of a conventional treatment process and the sampling locations of this study.

Samples were taken from the unchlorinated raw water, clarifier surface, clarifier sludge, filter surface, filter backwash, and finished water of the four plants. A schematic is shown in Figure 7. Generally, samples were collected from sampling taps available inside the plants though a pump to retrieve clarifier sludge samples and a sampling scoop to collect filter and clarifier surface samples were needed at some of the plants. Two 250 mL amber glass bottles were used to store duplicate samples for enzyme-linked immunosorbent assay (ELISA) analysis of saxitoxin and anatoxin-a and two 250 mL plastic PETG bottles were used for ELISA analysis of microcystins, cylindrospermopsin, and BMAA to avoid adsorption of the toxins to the storage vessels (Abraxis, Warminster, PA, USA). Two 500 mL amber glass bottles were used to store samples for cell enumeration. General water quality parameters were measured or obtained from the plant records as available and are presented in the Supplementary Information (Table S2).

### 4.3. YSI EXO2 Monitoring Probe

A YSI EXO2 Multiparameter Sonde (YSI, Yellow Springs, OH, USA) equipped with the Total Algae sensor for measuring phycocyanin in relative fluorescence units (RFU) was used in the 2017 algae season. The sensor was calibrated throughout the sampling season with rhodamine WT dye and was found to remain stable. 

The probe was used to measure the phycocyanin fluorescence of samples collected across the treatment trains. The fluorescence of the raw water, clarifier and filter surface, and finished water was recorded at each sampling visit. Clarifier sludge and filter backwash samples were not measured due to interferences caused by excessive turbidity. Triplicate readings were averaged to provide a single phycocyanin value for each sample. Coefficients of variation among the triplicates were generally below 0.2 but were higher for samples with very low fluorescence. 

### 4.4. ELISA Analysis

The concentrations of dissolved and total toxins were determined using the ELISA kits. Samples for dissolved cyanotoxin measurements were filtered through 0.2 µm syringe filters (Sarstedt Filtropur) to remove intact cyanobacteria cells and then kept frozen at −20 °C. Samples for total cyanotoxins (intracellular plus extracellular) were subjected to three freeze-and-thaw cycles to lyse the intact cells and release the intracellular toxins. Duplicate values were typically within about 20% but could have a difference of up to 50%. Absorbance was measured using an absorbance reader (Sunrise, Tecan Trading AG, Mannedorf, Switzerland). Online software [44] was used to produce a four-parameter logistic curve to calculate the cyanotoxin concentration of the samples. The method detection limits (Table S3), were generally higher than the manufacturer listed MDLs (Table S4), except for saxitoxin, which was comparable. Failure to recover most of the control standards in three of the five BMAA kits prevented the calculation of an MDL for those tests and their results are not reported.

Notwithstanding the uncertainty involved with and variable performance of the five ELISA kits in numerous studies [38,39,45,46,47,48,49], they were selected for such practical considerations as availability for the desired analytes and ease of use. 

The anti-ADDA microcystins ELISA kit measures all microcystin variants with the ADDA moiety except conjugated microcystins, though overestimation can occur in drinking water samples because of the presence of ADDA-containing chlorination by-products [50,51,52]. The anatoxin-a and cylindrospermopsin ELISA kits appear to demonstrate higher accuracy based on previous studies [45,53,54]. On the other hand, the saxitoxin ELISA kit is functional only from a presence/absence perspective [45]. Lastly, Faassen et al. [39] reported many likely false positives with the BMAA ELISA kit, but more recently, Clausi et al. [38] reported spiked recoveries in the range of 70–83%, which is adequate. Because this study is the first exploration of these five cyanotoxins across treatment trains in the drinking water plants, the weaknesses in the ELISA measurements were accepted but must be considered in the data interpretation. 

### 4.5. LC-MS/MS Analysis

A liquid chromatography-triple quadrupole mass spectrometry (LC-MS/MS) system (Agilent 6460 Triple Quadrupole LC/MS) was used to determine the microcystin-LR portion of the microcystins concentration reported by ELISA. The LC-MS/MS samples were prepared with an injection of 1 µg/L of nodularin as an internal standard. The method used a 0.5% formic acid acetonitrile and 0.5% formic acid water gradient through a C18 column (Agilent Poroshell 120 EC-C18). A positive control was included every 10 samples. The two 5 µg/mL stock solutions of microcystin-LR (Cedarlane Labs, Burlington, ON, Canada) and nodularin (BioLynx Inc., Brockville, ON, Canada) were stored at −20 °C in methanol and used to prepare the calibration standards.

### 4.6. Cell Microscopy

All the samples were preserved in Lugol’s iodine solution and enumerated by Lucja Heintsch Microscopy (Toronto, ON, Canada) to determine cell counts, species composition, and biovolume. Algae were identified on a Nikon inverted microscope at 100× − 600× magnifications. Biomass estimates were based on cell measurements, following the procedures listed in the Ontario Ministry of the Environment Phytoplankton Methods Manual. Because of the potential for significant differences in microscopy results between laboratories and as well as between individuals [55], one microscopist was retained for all sample analysis.

## Figures and Tables

**Table 1:** Treatment trains of the four drinking water treatment plants studied.

	Plant A	Plant B	Plant C	Plant D
**Treatment Train**	Prechlorination	Prechlorination		
	Coagulation/flocculation	Coagulation/flocculation	Coagulation/flocculation	Coagulation/flocculation
	Upflow clarification	Sedimentation	Sedimentation	Sedimentation
	Anthracite/sand filtration	Anthracite/sand filtration	GAC/sand filtration	Anthracite/sand filtration
	Chlorination	Chlorination	Chlorination	Chlorination

## Supplementary Materials

The following are available online at http://www.mdpi.com/2072-6651/10/11/430/s1, Figure S1: No correlation was found between cyanobacteria biovolume and phycocyanin fluorescence, Table S1: Cell speciation in all samples with concentrations in cells/mL, Table S2: General water qualities, Table S3: Method detection limits for all ELISA tests performed in µg/L, Table S4: Method detection limits for the ELISA test kits provided by Abraxis.
