# Supplementary material for: Cyanotoxins and Cyanobacteria Cell Accumulations in Drinking Water Treatment Plants with a Low Risk of Bloom Formation at the Source
Source: Toxins (Basel). 2018 Oct 26;10(11):430. doi: 10.3390/toxins10110430 (PMC6266306; doi:10.3390/toxins10110430)
Supplement: Supplementary file 1 [file toxins-10-00430-s001.pdf]

Supplementary Materials: Cyanotoxins and Cyanobacteria Cell Accumulations in Drinking Water Treatment Plants with a Low Risk of Bloom Formation at the Source

Husein Almuhtaram, Yijing Cui, Arash Zamyadi and Ron Hofmann

Table S1. Cell speciation in all samples with concentrations in cells/mL.

			**Microcystis**						**Aphan.**	**Coelos.**	**Woro.**	**Anabaena**				
			aeru.	sp.	wesenb.	viridis	novaceki	flos-aquae	flos-aquae	kuetz.	naegel.	sp.	circinalis	lemm.	spiroides	planctonica
Plant A		16-10-11	107	481					3	162		58	10		1	
Plant A		17-08-21	509	293	20		52			22						
Plant A	Raw water	17-10-02		69672					2	0.2			15			
Plant A		17-10-26	68	28		3	75								7	
Plant A		17-11-15		297												
Plant A		16-10-11		21					9	11						
Plant A		17-08-21		2583												
Plant A	Clarifier surface	17-10-02		5712												
Plant A		17-10-26		47												
Plant A		17-11-15		29												
Plant A		16-10-11		420	1029	200	6129	2245	197			102	239		24	
Plant A		17-08-21	539	5934												
Plant A	Clarifier sludge	17-10-02	105	6109	506	20		493								
Plant A		17-10-26	25	22514			554									
Plant A		17-11-15				257										
Plant A		16-10-11							1	21		11	1	1		
Plant A		17-08-21	2246	2158	851				112				81		12	
Plant A	Filter surface	17-10-02	216	5428	45											6
Plant A		17-10-26	75	118												
Plant A		17-11-15		52			119									
Plant A		16-10-11		234					68	537			73			
Plant A		17-08-21		238666	450				31				56			
Plant A	Filter backwash	17-10-02		113488												
Plant A		17-10-26	350	6501												
Plant A		17-11-15	10139	438												
Plant A		17-08-21														
Plant A	Finished water	17-10-02		341					0.3							
Plant A		17-10-26		24												
Plant A		17-11-15		4												
Plant B		16-09-27					7			13						
Plant B	Raw water	17-08-25														
Plant B		17-09-13														
Plant B		17-10-24														
Plant B		16-09-27								54		11		1	1	
Plant B	Clarifier surface	17-08-25											0.25			
Plant B		17-09-13											0.2	10		
Plant B		17-10-24	96	175						10						
Plant B		16-09-27														
Plant B	Clarifier sludge	17-08-25														
Plant B		17-09-13												22		
Plant B		17-10-24		16				820		10						58
Plant B		16-09-27								29			2	1		
Plant B	Filter surface	17-08-25				43										
Plant B		17-09-13												5		
Plant B		17-10-24												3		

Plant B		16-09-27										5	8			
Plant B	Filter backwash	17-08-25			2											
Plant B		17-09-13											18	6		
Plant B		17-10-24							3							4
Plant B		17-08-25														
Plant B	Finished water	17-09-13														
Plant B		17-10-24														
Plant C		17-09-07	424	140	143	21										
Plant C	Raw water	17-10-04	4		8			90	2						4	
Plant C		17-10-25	4			9		13	1							
Plant C		17-11-16	56					988	11	223						
Plant C		17-09-07	4	2			38									
Plant C	Clarifier surface	17-10-04	2												3	
Plant C		17-10-25	5	20	10										1	
Plant C		17-11-16	9													
Plant C		17-09-07	324	136797			260									
Plant C	Clarifier sludge	17-10-04		87												
Plant C		17-10-25	921		157											
Plant C		17-11-16					138									
Plant C		17-09-07			29											
Plant C	Filter surface	17-10-04		23											7	
Plant C		17-10-25				12										
Plant C		17-11-16														
Plant C		17-09-07														
Plant C	Filter backwash	17-10-04		28	462											
Plant C		17-10-25			83										2	
Plant C		17-11-16														
Plant C		17-09-07														
Plant C	Finished water	17-10-04														
Plant C		17-10-25														
Plant C		17-11-16														
Plant D		16-10-12								256						
Plant D		17-08-28	2	92					7							
Plant D	Raw water	17-09-08														
Plant D		17-10-06														
Plant D		17-11-23														
Plant D		16-10-12		0.1												
Plant D		17-08-28		12												4
Plant D	Clarifier surface	17-09-08														
Plant D		17-10-06														
Plant D		17-11-23														
Plant D		16-10-12		1300						657						
Plant D		17-08-28														
Plant D	Clarifier sludge	17-09-08														
Plant D		17-10-06							22	23						
Plant D		17-11-23		56												
Plant D		16-10-12														
Plant D		17-08-28		49												
Plant D	Filter surface	17-09-08														
Plant D		17-10-06														
Plant D		17-11-23														
Plant D		16-10-12	1065	35			200								52	
Plant D		17-08-28		209		34										
Plant D	Filter backwash	17-09-08		24												
Plant D		17-10-06														
Plant D		17-11-23														
Plant D	Finished water	17-08-28														
Plant D		17-09-08														

Plant D	17-10-06
Plant D	17-11-23

Table S2. General water qualities.

	Sampling visit																	
	Plant A					Plant B				Plant C				Plant D				
**Raw Water**	1	2	3	4	5	1	2	3	4	1	2	3	4	1	2	3	4	5
Temperature (°C)	19	24	21	14	12	-	6	13	8	22	20	17	11	19	24	21	22	12
Turbidity (NTU)	5.2	8.6	-	35	21	2.3	0.2	0.2	0.2	5	3.7	3.3	8.2	1.75	3.2	0.4	-	5.1
pH	8.2	8.6	-	-	-	8.2	7.4	-	7.7	8	7.8	8	0.9	8	8	7.5	7.7	7.7
Free Cl_2 (mg/L)	-	0	-	0	0	-	0.02	0.2	0	0	0	0	0	0.04	0.04	0.01	0	0
Total Cl_2 (mg/L)	0.01	0	-	0	0	-	0.01	0.34	0	0	0	0	0	0.07	0.03	0.01	0	0
DOC (mg/L)	2.1	3.3	3.3	1.8	1.9	2.6	2.3	0.2	2.1	16	6.2	5.3	5.9	2.6	2.9	6.8	2.7	2.3
**Finished Water**	1	2	3	4	5	1	2	3	4	1	2	3	4	1	2	3	4	5
Temperature (°C)	-	24	20	-	-	-	9	15	11	21	20	17	12	-	23	22	20	10
Turbidity (NTU)	-	0	-	0.05	0.06	-	0.1	0.1	0.05	0.2	0.3	0.3	0.3	-	0.1	0.2	-	0.1
pH	-	7.7	-	7.6	7.5	-	7.5	-	-	7.4	7.3	7.2	7.2	-	7.2	7.4	7.3	7.3
Free Cl_2 (mg/L)	-	0.12	-	0.05	0.08	-	0.05	0.05	0.07	2.3	2.5	2.4	2.7	-	1.2	1.2	1.4	1.3
Total Cl_2 (mg/L)	-	1.3	-	2.1	2.0	-	2.1	1.9	2.0	2.7	2.8	2.7	3.1	-	1.4	2.1	1.5	1.5
DOC (mg/L)	-	2.1	2.1	1.1	1.3	-	2.2	6.7	1.8	5.7	2.6	2.2	2.8	-	2.1	5.5	2.1	1.8

Table S3. Method detection limits for all ELISA tests performed in µg/L.

		Sampling visit				
**Cyanotoxin**		**1**	**2**	**3**	**4**	**5**
**Microcystins**	Plant A	0.37	0.75	0.39	1.07	0.33
	Plant B	0.37	0.75	0.43	1.07	
	Plant C	0.43	0.39	1.07	0.33	
	Plant D	0.37	0.75	0.43	0.39	0.33
**Anatoxin-a**	Plant A	0.27	0.60	0.21	0.24	0.16
	Plant B	0.27	0.60	0.18	0.24	
	Plant C	0.18	0.21	0.24	0.16	
	Plant D	0.27	0.60	0.18	0.21	0.16
**Saxitoxin**	Plant A	0.12	0.03	0.02	0.02	0.04
	Plant B	0.12	0.03	0.05	0.02	
	Plant C	0.05	0.02	0.02	0.04	
	Plant D	0.12	0.03	0.05	0.02	0.04
**Cylindrospermopsin**	Plant A	0.4	0.35	0.13	0.12	0.87
	Plant B	0.4	0.35	0.43	0.12	
	Plant C	0.43	0.13	0.12	0.87	
	Plant D	0.4	0.35	0.43	0.13	0.87
**BMAA**	Plant A	17.3	-	3.85	-	-
	Plant B	17.3	-	5.56	-	
	Plant C					
	Plant D	17.3	-	5.56	3.85	-

Table S4. Method detection limits for the ELISA test kits provided by Abraxis.

**Cyanotoxin**	**Manufacturer listed MDL (µg/L)**
**Microcystins**	0.1
**Anatoxin-a**	0.1
**Saxitoxin**	0.015
**Cylindrospermopsin**	0.04
**BMAA**	4

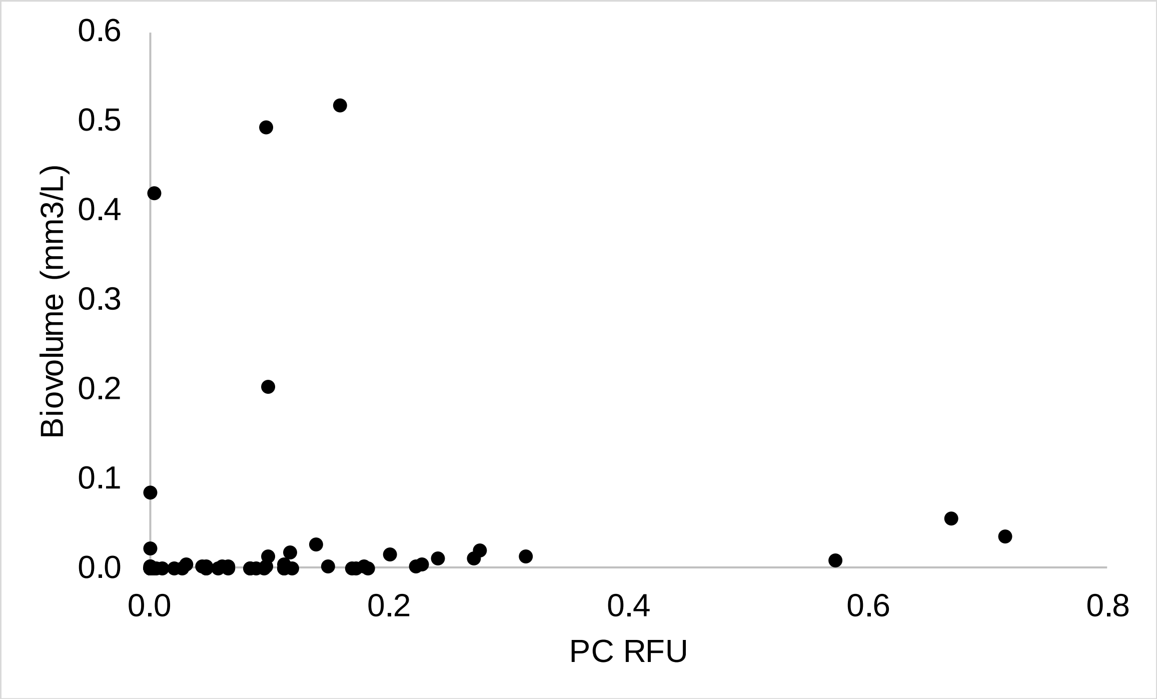

Figure S1. No correlation was found between cyanobacteria biovolume and phycocyanin fluorescence.
